# Child play and caregiver support to promote convalescence following severe acute malnutrition in Zimbabwe: The Tamba‐SAM pilot study

**DOI:** 10.1111/mcn.13726

**Published:** 2024-09-13

**Authors:** Jacqueline Kabongo, Louisa Mudawarima, Florence D. Majo, Anesu Dzikiti, Joice Tome, Bernard Chasekwa, Batsirai Mutasa, Lloyd Dzapasi, Epiphania Munetsi, Isabella Cordani, Robert Ntozini, Lisa F. Langhaug, Mutsa Bwakura‐Dangarembizi, Andrew J. Prendergast

**Affiliations:** ^1^ Zvitambo Institute for Maternal and Child Health Research Harare Zimbabwe; ^2^ Department of Paediatrics and Child Health, College of Health Sciences University of Zimbabwe Harare Zimbabwe; ^3^ Friendship Bench Trust Harare Zimbabwe; ^4^ Blizard Institute Queen Mary University of London London UK

**Keywords:** convalescence, malnutrition, mental health, play, psychosocial support

## Abstract

Children hospitalised for severe acute malnutrition (SAM) have a high risk of mortality, relapse and rehospitalisation following hospital discharge. Current approaches fail to promote convalescence, or to address the underlying social determinants of SAM, meaning that restoration of long‐term health, growth and neurodevelopment is not achieved. Although guidelines recommend play and stimulation to promote recovery, most caregivers are not supported to do this at home. We set out to evaluate the feasibility and acceptability of a codesigned intervention package aimed at providing child stimulation through play, and strengthening caregiver capabilities through problem‐solving skills, peer support and income‐generating activities. We evaluated the intervention in two phases, enroling 30 caregiver–child pairs from paediatric wards in Harare, Zimbabwe, once children who had been hospitalised with SAM were ready for discharge. Children were median 17.8 months old, and 28.6% had human immunodeficiency virus. Trained intervention facilitators (IFs)—lay workers whose own children had previously had SAM—delivered the intervention over 12 weeks with nurse supervision. Qualitative interviews with caregivers and IFs showed that the intervention was feasible and acceptable. Participants reported benefiting from the psychosocial support and counselling, and several started income‐generating projects. Caregivers appreciated the concept of play and caregiver–child interaction, and all reported practising what they had learned. By Week 12, caregiver mental health and caregiver–child interaction improved significantly. Overall, the intervention was feasible, acceptable and showed promise in modifying caregiver knowledge, attitudes and practice. An efficacy trial is now needed to evaluate whether the intervention can improve child convalescence following complicated SAM.

## Key messages

- A psychosocial intervention which enhances child play and promotes caregiver support may improve convalescence following severe acute malnutrition.
- We co‐designed a complex intervention, designed to provide child stimulation through play, and to strengthen caregiver and household capabilities through problem‐solving skills, peer support and income‐generating activities.
- The 12‐week intervention, delivered by trained lay workers whose own children had previously had SAM, was feasible and acceptable, and caregivers showed improvements in mental health and caregiver‐child interaction.
- This psychosocial support package should now be tested in a randomised trial to evaluate its efficacy in promoting convalescence.

## Graphical abstract

We codesigned an intervention package for children recovering from complicated severe acute malnutrition (SAM) in Zimbabwe, providing child stimulation through play, and strengthening caregiver capabilities through problem‐solving skills, peer support and income‐generating activities. The package was delivered by trained lay workers and was feasible and acceptable to caregivers, providing a potential new strategy to holistically enhance the home psychosocial environment during SAM convalescence.

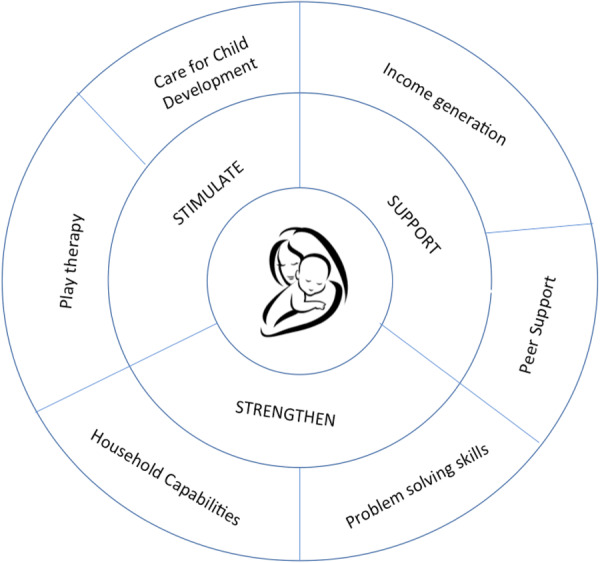

## 1 INTRODUCTION

Malnutrition underlies almost half of all child mortality (Black et al., 2013). Children hospitalised for severe acute malnutrition (SAM), the most life‐threatening form of undernutrition, have a high risk of mortality, relapse and rehospitalisation. In Zimbabwe, 9.1% of children died (Bwakura‐Dangarembizi et al., 2021) and 15.4% were readmitted in the year following hospital discharge (Bwakura‐Dangarembizi et al., 2022), with the highest risks among those with human immunodeficiency virus (HIV), nonoedematous SAM or cerebral palsy (Bwakura‐Dangarembizi et al., 2021). World Health Organization (WHO) guidelines recommend hospital discharge before full nutritional recovery (WHO Guideline on the Prevention and Management of Wasting and Nutritional Oedema (Acute Malnutrition) in Infants and Children under 5 Years, 2023) and children return to the same social and economic environment where SAM developed. Current approaches therefore fail to promote effective convalescence or to address the underlying social determinants of SAM, meaning that restoration of long‐term health, growth and neurodevelopment is not achieved.

There is scant evidence on strategies to promote convalescence among children with SAM. A recent meta‐analysis demonstrated that child play and stimulation in the community reduced mortality and improved neurodevelopment (Noble et al., 2021). Although WHO guidelines recommend play and stimulation (WHO Guideline on the Prevention and Management of Wasting and Nutritional Oedema (Acute Malnutrition) in Infants and Children under 5 Years, 2023), there is no formalised way to deliver and sustain the intervention at home. Children with SAM are frequently discharged to a home environment characterised by poverty and multiple caregiver vulnerabilities including low decision‐making autonomy, lack of social support, gender‐restricted family relations and competing demands on scarce resources (Zakayo et al., 2020). Caregiver depression is common in families affected by SAM (Nguyen et al., 2014; Stewart et al., 2011) and may reduce interaction with the child. Developing a complex intervention to enhance caregiver support, promote household income generation and encourage caregiver–child interaction may stimulate child development and foster recovery following SAM. We therefore assessed the feasibility and acceptability of a child play and caregiver support package to enhance the psychosocial environment during convalescence.

## 2 METHODS

### 2.1 Intervention theory

**Figure 1:**
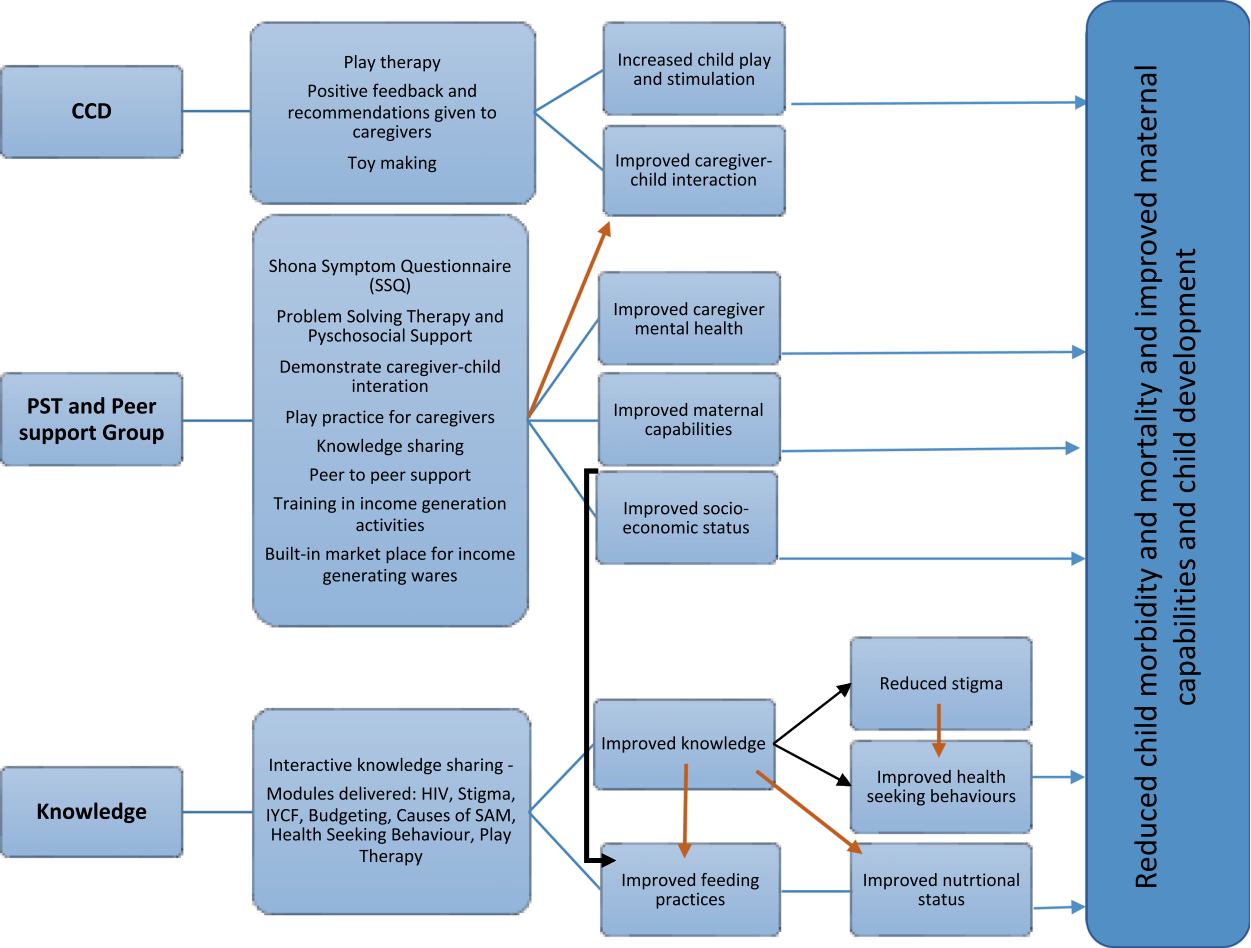
Theory of change. The study's theory of change has its underpinnings in the UNICEF Conceptual Framework on Maternal and Child Nutrition and the WHO Nurturing Care for Early Childhood Development Framework. The intervention package combined the UNICEF Care for Child Development (CCD), which provides stimulation for the child through play therapy; the Friendship Bench, which provides problem‐solving therapy (PST) for the caregiver, with peer support and income‐generating activities; and modules to increase the knowledge of the caregiver. Observing caregiver–child interaction, giving positive feedback and recommendations to caregivers, and teaching them how to make toys using locally available resources, increases stimulation for the child and improves interaction and emotional bonding with the caregiver. PST strengthens caregiver and household capabilities through problem‐solving skills, while CKT groups work by providing peer support and engaging in income‐generating activities. Increasing the caregiver's knowledge through targeted interactive knowledge‐sharing activities increases caregiver capabilities, improves feeding practices and health care‐seeking behaviours. The overall goal of the intervention package is to reduce child morbidity (all‐cause readmissions) and mortality, and improve maternal capabilities. CKT, Circle Kubatana Tose; WHO, World Health Organization.

We first conducted a systematic review, which identified child play and stimulation as evidence‐based interventions to improve outcomes following hospitalisation with SAM (Noble et al., 2021). Next, we developed a theory of change (Figure 1) based on the UNICEF Conceptual Framework on Maternal and Child Nutrition (Unicef Conceptual Framework on Maternal and Child Nutrition, 2021) and the WHO Nurturing Care for Early Childhood Development Framework (WHO, 2018). We focused on factors underlying undernutrition at the household level, since children are discharged back to the same home environment where they developed SAM, and caregivers often have multiple vulnerabilities. We reasoned that a package addressing the combined needs of the child–caregiver pair could holistically enhance convalescence.

The goal of the intervention was to provide stimulation for the child through play and to strengthen caregiver and household capabilities through problem‐solving skills, peer support and income‐generating activities. We selected two core components based on the existing literature and local applicability. The Friendship Bench was developed in Zimbabwe to provide problem‐solving therapy through lay workers, to treat common mental disorders (Chibanda et al., 2015). UNICEF Care for Child Development (2019) is a UNICEF package that helps families build stronger relationships and solve problems in caring for their child, through age‐specific play and communication activities. We leveraged existing training materials and tools for both interventions and devised an integrated series of home visits to deliver the combined intervention, which we termed ‘Tamba‐SAM’ (meaning Play‐SAM in Shona).

### 2.2 Intervention development and evaluation

Our aim was to pilot the intervention, modify it through codesign, and evaluate feasibility and acceptability using mixed methods. We used an Experience‐Based Co‐Design (EBCD) approach, which brings together patient and provider experiences to improve services and care (Fylan et al., 2021; Macdonald et al., 2023). The evaluation was conducted in three phases: first, the intervention (‘Tamba‐SAM 1.0’) was delivered to 20 caregiver–child pairs for 12 weeks; second, these caregivers joined codesign workshops to modify the intervention based on their experiences; and third, the refined intervention (‘Tamba‐SAM 2.0’) was delivered to 10 new caregiver–child pairs. The protocol, intervention materials, and case report forms are available at https://osf.io/4py8z/. We followed recommended reporting guidelines for implementation research on nurturing care interventions designed to promote early childhood development (Yousafzai et al., 2018).

#### 2.2.1 Intervention facilitators (IFs)

We selected and trained a group of caregivers whose own children had previously had SAM, to become lay peer counsellors, whom we termed IFs. IFs had to have literacy of the local language (Shona) and secondary school education. They were selected during an interview process, which included activities with volunteer caregivers and children to assess their ability to interact and play with children. IFs received an allowance and transport reimbursement based on the number of days worked.

The IFs were trained by the research team, and the Friendship Bench team, which entailed theory and clinical practice for 10 days, followed by practice sessions and simulations for a further 10 days. During the course of the study, they received continued support and supervision through weekly review of audio/video recordings and debriefing sessions. The Friendship Bench team supervised the initial CKT sessions and liaised with community members to share income‐generating ideas.

#### 2.2.2 Tamba SAM 1.0

**Figure 2:**
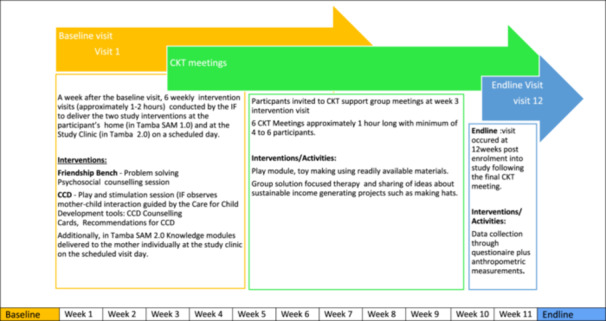
Study design. The Tamba‐SAM study was conducted in two phases: initially as Tamba‐SAM 1.0, and then as Tamba‐SAM 2.0 following refinement. In both phases, caregiver–child pairs were in the study for 12 weeks in total. Following a baseline data collection visit by a research nurse, intervention facilitators started the intervention, which combined problem‐solving therapy through the Friendship Bench and child play through the Care for Child Development (CCD); in Tamba‐SAM 2.0, additional educational modules were integrated into the visit. After at least three individual sessions, the caregiver was referred to a Circle Kubatana Tose (CKT, ‘holding hands together’) peer support group. At Week 12, an endline visit by a research nurse was undertaken to collect outcome data. SAM, severe acute malnutrition.

The study design is shown in Figure 2. Participants were recruited from the paediatric wards at Sally Mugabe Hospital in Harare, Zimbabwe, which serves a predominantly peri‐urban community. The study population comprised children aged 6−59 months, hospitalised with SAM, who were clinically stable and preparing for discharge, with a primary caregiver willing to receive home visits. Children discharged to a home outside greater Harare were ineligible. All families received the intervention; there was no control group.

A research nurse conducted a baseline home visit within 7 days postdischarge. Data were collected on caregiver demographics, socioeconomic status, education, religion, and household dietary diversity. Caregiver depression was evaluated using the Edinburgh Postnatal Depression Scale—a 10‐item self‐report questionnaire validated as a screening tool for depression (Cox, 1996). Each item is scored from 0 to 3 and a total score of 12 or more, or an affirmative response to a question on self‐harm, indicated a high risk for depression. Caregiver alcohol use was evaluated using the Alcohol Use Disorders Identification Test (WHO, 2001). Gender norms and social support were assessed using quantitative tools (Tome et al., 2021). Children had weight, height, mid‐upper arm circumference and head circumference measured and development assessed using the caregiver‐reported Ages & Stages Questionnaire (ASQ). Responsive caregiving was assessed using the Observation of Maternal‐Child Interaction (OMCI), which measures mother–child interactions (Rasheed & Yousafzai, 2015). The mother and child are given a locally designed story book and observed for 5 min. The assessor undertakes scoring using a maternal and infant interaction framework (Landry et al., 2006), which comprises 12 maternal behaviour items, six child observation items and one mutual enjoyment item. Maternal behaviours assessed include touch, affect, verbal statements and language stimulation; child behaviours include attention, communication and affect. Each item is scored from 0 to 3 (0 = never observed, 3 = observed over five times) with a maximum score of 57. A higher score indicates a more responsive interaction.

The intervention was delivered to the caregiver–child pair weekly over 6 weeks through home visits from the IFs. Intervention visits lasted 1–2 h and were audio‐recorded with consent. A nurse supervisor attended sessions to observe intervention delivery, which were video‐recorded with consent, to assess fidelity. Fidelity was defined as the degree to which the intervention was implemented as intended (Proctor et al., 2011), that is, the extent to which the IFs adhered to the delivery protocols and components of the Tamba SAM package. It was assessed using process indicators (number of sessions delivered, content of sessions), qualitative data from the visit outcome form about whether the correct steps were followed and modules delivered and review of video and audio recordings. After at least three individual sessions, the caregiver was referred to a Circle Kubatana Tose (CKT, ‘holding hands together’) support group. CKT groups, which bring women together to share and solve problems, and to initiate income‐generating activities, were facilitated by an IF with supportive supervision from a research nurse. CKT groups comprised 4–6 participants, lasted for approximately 1 h, and were delivered weekly for 6 weeks; the total intervention package therefore lasted 12 weeks. A home visit was conducted by the research nurse 12 weeks postenrolment (window 12–16 weeks) to collect endline data.

#### 2.2.3 Codesign workshops

After the initial pilot, caregivers and IFs were invited to two codesign workshops. We also invited a group of grandmothers from the same communities, since grandmothers have been identified as influential in decision‐making around child feeding and health‐seeking behaviours in Zimbabwe, as in many parts of sub‐Saharan Africa (Karmacharya et al., 2017; Michel et al., 2020; Reddy et al., 2022). Feedback identified knowledge and information gaps among caregivers. We therefore developed six modules on (i) causes of SAM, (ii) feeding an adequate diet, (iii) feeding a child during illness, (iv) finding help for a sick child, (v) stigma and (vi) budgeting. A six‐part series of modules on HIV was also developed, and the modules were delivered weekly by the IF during the individual sessions with the caregiver. Finally, a play and stimulation module was designed for CKT sessions. We integrated the new material into the existing intervention to create a refined package termed Tamba‐SAM 2.0 (Figure 2).

#### 2.2.4 Tamba‐SAM 2.0

Ten new caregiver–child pairs were enroled using the same inclusion criteria. In Tamba‐SAM 2.0, intervention visits occurred at the hospital, since caregivers had expressed challenges in receiving home visits due to lack of privacy, gossip from neighbours and unwillingness among landlords to allow visitors. Caregivers received transport reimbursement to attend visits.

### 2.3 Data analysis

#### 2.3.1 Quantitative analysis

Outcomes were assessed at baseline and endline for each caregiver–child pair. Pre‐ and postintervention continuous measures were compared using paired *t*‐tests if normally distributed or Wilcoxon signed‐rank tests if non‐normally distributed. Proportions were compared using McNemar's test. Only matched pairs were included in the analysis. All quantitative analyses used Stata version 14 (StataCorp LLC).

#### 2.3.2 Qualitative analysis

Primary data sources included baseline and endline surveys, visit outcome forms, audio recordings of intervention visits, notes from CKT meetings, IF debrief sessions and supervisory visits. Audio recordings were transcribed and translated. Data were analysed using thematic analysis. A coding framework with main codes and sub‐themes was developed through iterative reading of transcripts and notes.

### 2.4 Ethical approvals

The Medical Research Council of Zimbabwe approved the study. Caregivers gave written informed consent to participate.

## 3 RESULTS

**Figure 3:**
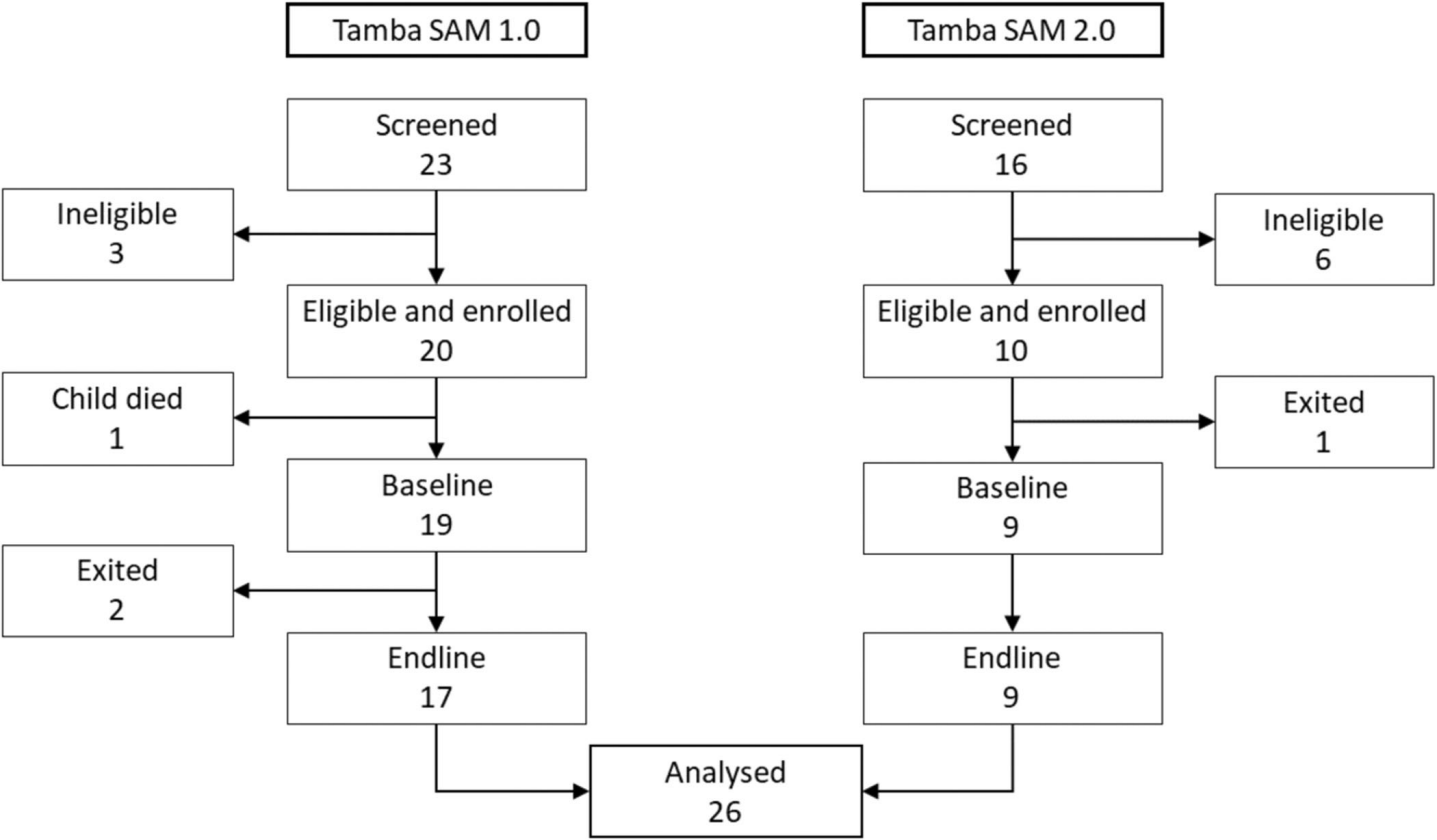
Participant flow. The pilot study was conducted in two phases: Tamba‐SAM 1.0 piloted the initial intervention, before refinement through codesign, then Tamba‐SAM 2.0 tested the modified package. Caregiver–child pairs were screened for eligibility on the paediatric wards of Sally Mugabe Hospital, Harare, and were invited to enrol if eligible. The baseline visit occurred within 7 days of the child leaving hospital and was conducted at the participant's home; one child died in Tamba‐SAM 1.0 between enrolment and baseline, and one caregiver–child pair exited in Tamba‐SAM before baseline. Two caregiver–child pairs exited between baseline and endline during Tamba‐SAM 1.0. A total of 26 caregiver–child pairs were evaluated at endline. SAM, severe acute malnutrition.

The study design is shown in Figure 2 and the participant flow in Figure 3. Of 20 caregiver–child pairs enroled into Tamba‐SAM 1.0, one child died before the baseline visit. During follow‐up, two caregiver–child pairs exited; 17 caregiver–child pairs were therefore evaluated at endline. Of 10 child–caregiver pairs enroled into Tamba‐SAM 2.0, one withdrew consent before the baseline visit; nine participants completed the study. Baseline characteristics are shown in Table 1. Children were median 17.8 (interquartile range [IQR] 12.5−22.7) months old; almost half (46.4%) had oedematous SAM and over one‐quarter (28.6%) had HIV. In all cases except one, the primary caregiver was the mother, with median duration of schooling of 10 years (IQR 9−11); only half were employed.

**Table 1:** Baseline characteristics.

Characteristic	All (*N* = 28)	Tamba‐SAM 1.0 (*N* = 19)	Tamba‐SAM 2.0 (*N* = 9)
*Child*			
Age, months, median (IQR)	17.8 (11.8−22.3)	20.0 (14.3−22.9)	10.1 (8.0−17.7)
Male sex, %	42.9	42.1	44.4
Oedematous SAM, %	46.4	36.8	66.7
WHZ, mean (SD)	−2.2 (1.4)	−2.3 (1.2)	−2.0 (1.9)
WAZ, mean (SD)	−3.6 (1.6)	−3.6 (1.4)	−3.4 (2.1)
HAZ, mean (SD)	−3.5 (1.4)	−3.6 (1.2)	−3.3 (1.9)
MUAC, mm; mean (SD)	118 (14)	118 (12)	117 (20)
HCZ, mean (SD)	−1.2 (2.2)	−1.4 (2.3)	−0.9 (2.2)
Stunted, %	89.3	94.7	77.8
Underweight, %	89.3	89.5	88.9
Living with HIV, %	28.6	26.3	33.3
*Caregiver*			
Mother of child, %	96.4	100.0	88.9
Employed, %	50.0	47.4	55.6
Age, years, median (IQR)	25.0 (23.0−32.0)	25.0 (23.0−33.0)	25.0 (23.0−30.0)
Schooling, years, median (IQR)	10.0 (9.0−11.0)	10.0 (9.0−11.0)	11.0 (9.0−11.0)
*Household*			
Improved sanitation, %	100.0	100.0	100.0
Electricity, %	39.3	42.1	33.3
Household size, mean (SD)	5.0 (2.2)	4.9 (2.0)	5.1 (2.6)
Household dietary diversity score, median (IQR)^a^	7.5 (6.0−8.0)	7.0 (6.0−8.0)	8.0 (7.0−8.0)

Abbreviations: HAZ, height‐for‐age *Z* score; HCZ, head circumference for age *Z* score; IQR, interquartile range; MUAC, mid‐upper arm circumference; SAM, severe acute malnutrition; SD, standard deviation; WAZ, weight‐for‐age *Z* score; WHZ, weight‐for‐height *Z* score.

^a^ Household dietary diversity is scored from 0–16, based on the number of food groups (out of 16) consumed by the household in the previous 24 h.

### 3.1 Feasibility of intervention delivery

Four IFs were successfully trained to deliver Tamba‐SAM 1.0. Among the 19 caregivers, 17 received all six problem‐solving sessions. Some landlords objected to home visits, citing COVID‐19 fears, overcrowding and disruption of normal routines. Ensuring privacy was difficult due to concerns about eavesdropping during intervention sessions. One participant expressed initial shame over the idea of home visits:

‘It was difficult for me at first because my house is very small… but I had to accept to participate because I needed a better outcome for my child’. (10008).

Thirteen caregivers completed all six CKT sessions; one completed five sessions, one completed two sessions, and two attended no sessions. Reasons for missing CKT sessions included challenges finding childcare, clashes between CKT and work time, attending funerals, caring for sick relatives, visiting rural home areas and failing to get transport.

In Tamba‐SAM 2.0, all participants received six problem‐solving therapy sessions. There was good fidelity to planned activities, with all components of the intervention successfully delivered. The IFs attributed this to the clearly structured, easy‐to‐follow tools and modules, and also the on‐going monitoring and mentorship. Overall, 28/161 (17%) visits had supportive supervision from a senior nurse, involving review of the visit outcome form, providing feedback on audio recordings or sitting in on sessions. IFs sometimes found that supervisory visits increased their anxiety, affected the flow of the session, or meant participants were less open to disclosing their problems; however, they appreciated the learning that arose from supervisory visits (Table 2, 1.1–1.2).

**Table 2:** Additional qualitative data from respondents.

*Intervention facilitators: Supportive supervision*	
1.1	‘When we had supervisory visits at times I felt nervous that I might be making a mistake. But I assured myself that since I had been trained on it, I will continue with the session with assurance that I will be corrected. The objective of supervision is good as one may continue… with some errors. Like in my case when I was counselled to involve the participant in the discussion [rather] than me lecturing to her’. (IF3)
1.2	‘I never had problems with the supervisor observed sessions, but my participants would become shy. I benefitted from the sit‐in, because the supervisor will highlight areas that needed attention and skills I should have employed as a learning process. It was good’. (IF1)
*Intervention facilitators: interaction with caregivers*	
1.3	‘…talking to someone who walked through what you are going through is so relieving’. (10004)
1.4	‘I found it very helpful in that at least I had someone to share my problems with and they were able to share one or two ideas. Like when I was being asked those questions on how I was feeling, if I was stressed out, I would be relieved after sharing. If I had come with a heavy head, I would go home with the load eased off. I also got ideas on how to move forward’. (10026)
1.5	‘You know that people in the neighbourhood just want to ask so they have something to laugh about, [they have] ‘ ‘itching ears,’ while here, these are trained people who can counsel and assist you. Those in the community will go about talking or gossiping about it. It was therefore easy for me [to talk to the IF] because they will not go around my neighbourhood talking about it’. (10026)
1.6	‘During PST counselling they were opening up regarding the stressful issues they were going through’. (IF3)
1.7	‘In the initial stages it was tough, but after building a relation of trust they opened up about their private life problems’. (IF1)
1.8	‘It used to be difficult for me but I got used to it… it was difficult and I was so embarrassed about my life and I was just afraid to talk about it’. (10029)
1.9	‘With the health seeking behaviour [module] some participants would give responses that will please the IF, but later on own up, with their beliefs in witchcraft as the cause of the child's illness, and about seeking help from faith healers’. (IF2)
*Income‐generating projects among participants*	
1.10	‘The IF was so relaxed and showed a lot of patience towards me…I shared with the IF how I was struggling to make ends meet. It was difficult for me to get enough food for my children. The IF encouraged me to start an income‐generating activity like selling food items such as vegetables so as to supplement my finances and be able to offer my family support. She would check on the progress each time I came for the visits and would encourage me not to lose hope. This really helped me and I eventually got financial assistance and managed to start the project of selling chair backs (covers). I am now able to buy my child fruits like bananas upon request or whenever I want to, unlike long ago when I would only buy for her if I had the money’. (10028)
1.11	‘The discussions I had with the IF made me bold and confident and able to stand up for myself. It also encouraged me to come up with ways of generating income so that I'm able to take care of the family’. (10025)
*Changes in childcare practices noted by intervention facilitators*	
1.12	‘Some [participants] owned up that they never knew that playing with the child stimulates the child's development and were now sharing the same information with others. Some had perceived fruits as expensive and resorted to snacks like commercial maheu (fermented maize drink)’. (IF1)
1.13	‘All my participants were able to practice what they had learnt, like starting income generating activities. Some of them are still chatting with us on the phone, telling us how well they are doing’. (IF2)
*Participant challenges*	
1.14	‘The doctors encouraged me to give a 4‐star diet as taught at the hospital. I will see how it goes but it's difficult for me to come up with the recommended diet… That is exactly what I am pointing out, that it's hard for me to get even a small amount of milk or butter to add to his diet… My child is failing to gain weight because of lack of money’. (10002)
*Peer support groups*	
1.15	‘I was happy to share experiences and exchange ideas about IYCF with other caregivers, especially about the mixed food recipes. We also shared our experiences on play therapy…ah it was really exciting. People would share their problems and we would manage to come up with solutions to the problems shared’. (10025)
1.16	‘CKT group sessions were good in that we were able to meet as mothers who had been admitted in hospital. We were able to share our experiences that led to the child's illness. In sharing…, one will be able to learn what caused the child to be sick, and would know how to prevent that from happening to you also. We also learned ideas from each other on the food recipes that are nutritious for the child’. (10026)
*Caregiver views on play*	
1.17	‘I also never used to care about the importance of caregiver and child interaction and I would ask my child to go play with other children but I have realised that playing with my child has helped her. She can now correctly name items and is now able to follow instructions…my child is now clever’. (10028)
1.18	‘Actually, I did not know that as a caregiver I should find time to play with my child and would just give him toys to play with alone. At the moment I am now finding time to play with my child. If he's playing with his toy car, I also get a toy car myself and I start to play with him. We also play various other games like ball games… There is a change these days; he is now calling me when he wants us to play, he says “Mama come let's play.” ’ (10029)
1.19	‘I used to think it was a waste of time to put aside time to play with my child. I can say I was just too lazy to do that. I also thought that my child was still very young and it would be better if she gets the time to play with other children of her age group… At times I would wonder what she would learn from me… I am seeing a great change; her brain is functioning in a better way and one might mistake her age thinking that she is someone about 5 years old. She is now clever’. (10028)
1.20	‘I enjoyed learning about the importance of child play. When I started learning about child play my child was still sick. At first, I felt my child was still too weak to be engaged in play activities but through the constant effort I started to see a change in my child. I thought my child would not be able to respond to the interaction and stimulation such as smiling back. I just continued to play with her and within no time she started to respond to the actions’. (10030)
1.21	‘I also used to be very impatient and harsh towards my child, but I now appreciate that I need to be gentle towards her. During feeding times my child would not eat much compared to what I expected and this would make me angry. It is through [these] lessons that I started to understand and appreciate that during illness a lot will be going on in the child's body, be it pain or loss of appetite. When I started practising responsive feeding, I saw a great change in my child’. (10030)
1.22	‘I would say the Tamba SAM programme helped in giving [me] knowledge of what we didn't know, like playing with our children tenderly without being harsh’. (10025)
1.23	‘My child's communication skills have improved. Previously she was not able to follow instructions, it would appear as if she was not hearing what I was telling her to do. For example, if I asked her to name objects, like, “show me the baby,” “show me the cup,” or “get the rattle" she was someone who did not know all these things and would not take any action. I have noted that playing and interacting with [name] has really helped and her communication and understanding has improved’. (10028)
1.24	‘I am seeing a great change in my child in terms of her growth and development. My child is now able to sit on her own, something that I never thought she would be able to do’. (10030)
1.25	‘I now appreciate that child play is not only about buying toys from the shops but as a caregiver, I can just make some toys using available material. During the CKT meetings I witnessed the various toys that the [other] caregivers had made for their children. I learned that I cannot mention lack of toys as something that can stop me from playing with my child because I have not managed to purchase a doll for her but from the lesson, I know I can simply make something to use during play’. (10030)
*Caregiver learning and intervention uptake*	
1.26	‘Ah! I just cared for them as children; cook porridge, add salt and sugar, and that's it, as long as they have had porridge. In Tamba SAM, I then learnt that you can add something to the porridge. You can add peanut butter, not on a daily basis, but alternate with margarine, kapenta, beans, milk, eggs or meat’. (10027)
1.27	‘I enjoyed most learning about IYCF. I lacked knowledge of basic things like proper ways of storing left‐over food. I am making sure that my child's food is stored in a clean container which is tightly closed. I am also reheating any leftovers before giving it to my child’. (10028)
1.28	‘I know they are not encouraged [honey pot maheu]. But I just wanted him to be full’. (10027)
1.29	‘…I also did not know how to prepare a proper meal for my child but since the Tamba SAM programme I now cook better and play well with my child. I was just used to cooking porridge. I would also buy him maheu (honey pot) and zapnax for lunch because he never used to like whatever I would have prepared for lunch, in the evenings I would buy him some more maheu and give it to him because he did not like the [other] food. He had a poor appetite and he preferred drinking maheu…I am now able to prepare my child's food in a better way using the mixed foods recipes’. (10025)
1.30	‘I used to eat all the meat and would at times just give my child a small portion of meat. Sharing our experiences as caregivers of children who were once admitted with SAM made me realise some of the things I was doing was out of ignorance’. (10028)
1.31	‘The snakes and ladders helped me to know the good and the bad habits, for example, when playing and the dice landed on a grid with dirty water. It was easy for me to reflect on what I would have done wrong, for example, giving my child dirty water’. (10025)
1.32	‘The game of trying to fill a jar that had holes with stones helped me because with [name], I would feed him and the next minute he had diarrhoea and vomiting. So nothing was being absorbed in the body to build it up or for him to grow. So the game helped me understand what had happened to my child’. (10023)
1.33	‘My child is now gaining weight because I learnt more on how to prepare his meals in a better way by mixing the right foods and mashing’. (10008)
1.34	‘I didn't know you can add all that in the porridge: avocado, matemba (kapenta) or egg or margarine, banana or milk…’ (10010)
1.35	‘When I don't have money to buy him eggs, I get a vegetable leaf, wash it, cut into small pieces… and add to the porridge…’ (10027)
1.36	‘Before Tamba SAM, I did not mind about taking the tablets at the prescribed time and would just take them haphazardly… I am now able to take my tablets on time as prescribed’. (10025)
1.37	‘The lesson on HIV also made me understand that being HIV‐positive is not the end of the world… I had lost all hope and thought that my child would not make it, but I am happy that my child is now showing great improvement’. (10030)
*Overall views on the Tamba SAM intervention*	
1.38	‘There was nothing I did not like; everything was good and enjoyable for me’. (10027)
1.39	‘…it will be ideal if they continue with this programme in a bid to help other caregivers who may have this problem’. (10030).
1.40	‘[If you were asked to repeat CKT meetings, would you want to?] Yes, I would want to, provided I have no other demands which may make it difficult for me to attend the CKT. Even if it means attending it for the whole year, I have no problem’. (10028)
*Sharing intervention learning with others*	
1.41	‘I will tell her that that this is a good programme and is very valuable…the programme taught me to take the child to the hospital when he is sick, the type of food to feed the child, to get tested for HIV even when you were negative before, know your status; I can tell someone all that’. (10027)
1.42	‘I have also shared with my neighbour, especially about IYCF and she was happy to learn from me… I have a friend whom I have already shared with about IYCF and I am discouraging her from giving her child junk food like maheu, zapnax, chips, etc. She took my advice and started to practice the information I shared with her’. (10028)

Abbreviations: CKT, Circle Kubatana Tose; IYCF, infant and young child feeding; SAM, severe acute malnutrition.

IFs successfully employed counselling skills in problem‐solving therapy and helped caregivers identify solutions to their problems. Participants reported that they benefited from the psychosocial support and counselling they received and appreciated having someone they could talk to about their problems (Table 2, 1.3–1.4). IFs found that once they shared their own lived experience as mothers of children with SAM, participants opened up:

‘A module like the [one on] health‐seeking behaviour was very interesting to me given I had lived the experience of seeking help from a faith healer when my child was sick. So, I could relate with the participant's way of thinking’. (IF2)

According to participants, the IFs were very open and made the caregivers feel comfortable sharing their problems. One caregiver mentioned that speaking to a trained stranger was better than friends and neighbours:

‘The IF was very open and this made me able to confide in her. I saw that she… was someone who would not laugh at me or point fingers at me or disclose about my HIV status to other people’. (10025; see also Table 2, 1−5).

Interviews with IFs showed that all participants were able to disclose personal issues (Table 2, 1.6–1.7). Some caregivers, who initially found it difficult to share problems, said they opened up more as rapport and trust were built (Table 2, 1.8–1.9).

IFs sometimes dealt with suicidal ideation among participants. All such cases were reported to their supervisor for assistance, and IFs played a critical role in counselling and supporting these caregivers. One participant expressed appreciation for how she was counselled to deal with her marital problems at a time when she was feeling suicidal:

‘I had problems with my husband, and I received counselling from the IF. Since then, I have not had suicidal thoughts as before…’ (10006)

The impact of psychosocial therapy was also felt by participant families. One participant highlighted that her husband had observed that she appeared more relaxed whenever the IF came for a session.

‘…having someone who comes in and they spend time discussing life issues with you is very helpful. My mind will be lighter. Even after the counselling session, my husband notices the lightened burden on my face…’ (10010)

Counselling by IFs helped to improve caregiver capabilities. One participant highlighted that her discussions with the IF were an ‘eye‐opener’ for her (‘kuvhurika pfungwa’), which led her to realise that she could start a business venture (a market stall), which is now helping meet some family expenses. Several other caregivers highlighted that counselling encouraged them to start an income‐generating project (Table 2, 1.10–1.11). IFs reported that all participants were receptive to the knowledge shared with them. They highlighted how caregiver perceptions about several childcare practices were changed (Table 2, 1.12–1.13).

The interviews and debrief sessions highlighted that self‐care for the IFs was important. The IFs described sometimes being affected by the stressful situations experienced by participants, and they were sometimes facing the same challenges themselves. Debrief sessions and journaling reflections helped IFs to cope with this stress.

### 3.2 Acceptability of intervention

#### 3.2.1 Problem‐solving therapy and peer support

Participants were mostly from vulnerable, food‐insecure families and financial challenges were the most common problems cited. Several participants lacked money for food to meet the recommended diet for both the child and the rest of the family. This was a main contributor to non‐adherence with dietary advice provided in hospital (Table 2, 1−14). The CKT meetings created a platform for participants to provide peer‐to‐peer psychosocial support and exchange ideas and advice on small businesses, play, toy‐making, and feeding practices (Table 2, 1.15–1.16). Solutions included starting, reviving, or expanding a business venture, or taking up part‐time domestic work. Participants devised income‐generating projects such as making patch‐blankets and quilts for sale, offering cellphone charging services, or selling fruit and vegetables, second‐hand clothes and other wares:

‘I got money from a friend of mine to start fruit vending, and eventually I want to have a tuck shop. I have been selling bananas and apples, and with the proceeds my sister is going to help me get a bale of clothes from South Africa around August. Now I even have a gas cooker’. (10027)

During CKT meetings, participants were taught how to crochet; one participant started making hats and bags to sell. This participant highlighted that she was excited at having learnt a skill that allowed her to generate money while working from home and spending time with her children.

#### 3.2.2 Play and caregiver–child interaction

While most participants were aware of the need for children to play, they did not view it as something they should be directly involved in as caregivers. However, by the end of the intervention, most caregivers appreciated the concept of play and caregiver–child interaction. They consciously made time to play with their children and were seeing the benefits. Several participants highlighted that they thought children play on their own or with other children (Table 2, 1.17–1.19). Several admitted that they believed their children were too young to understand or too sick to play:

‘I don't know why, but when I looked at my child, I thought that he was still very young and not able to follow or imitate my actions. But the moment you talked about the importance of play I started practising it and wanted to see if it really works. It has worked; my child is now clever’. (10029); see also Table 2, 1.20.

Another two participants shared that they learnt not to be harsh with children (Table 2, 1.21–1.22). One caregiver whose child had cerebral palsy said the intervention helped shift the negative perceptions she had about the child's disability and she had started to play with her child and could see improvements:

‘Before, I used to say, “how do I play with a child who does not know anything at all?”; but now when I say to her, “look at that insect,” she actually looks around for it. I can tell she is learning’. (10010)

All participants reported seeing improvements in their children following the intervention. Several caregivers reported specific improvements in gross and fine motor skills or improved communication:

‘Before Tamba‐SAM [name] would just stare at the ball or bite it, but since I started interacting and playing with him, he is now able to play with the ball, throw it or even follow it to pick it up. My child is now able to imitate me; for example, when I sing a song, he sings along with me… This is something he never used to do. I then see that his brain is functioning well from all that’. (10029); see also Table 2, 1.23–1.24.

All caregivers were innovative in making toys at home using available items such as used paper and plastics to make a ball; a rattle made from metal bottle tops; and containers used as musical instruments. This dispelled the myth that toys had to be bought and were thus unaffordable. One caregiver made a colourful picture chart for her child, and other caregivers were motivated to make similar toys for their children (Table 2, 1.25).

#### 3.2.3 Interactive behaviour‐change modules

Most participants stated that the modules highlighted areas where they had been undertaking inappropriate practices, such as food‐handling, hygiene, meal frequency and nutrient content of meals:

‘I learnt that you should not just feed for the sake of feeding, but that she should have all nutrients; carbohydrates and proteins. I never used to consider it as something important’. (10026); see also Table 2, 1.26–1.27.

Some participants admitted feeding their children unhealthy snacks out of convenience because the child was a fussy eater or had poor appetite, and they wanted the child to be full (Table 2, 1.28–1.29). Other participants mentioned that they used to feed their children based on myths and misconceptions about what children can and cannot eat, but learnt the correct feeding practices through the intervention:

‘We were instructed on the correct way of feeding the child. We didn't know about the amounts; we would just feed porridge with peanut butter then later feed sadza with soup. We didn't give fish for fear the child would choke on bones. We did not give meat believing that the child will become greedy, or fruit because he will steal fruits from other people's orchards, or the child will cry for them when you don't have money to buy any…’ (10022); see also Table 2, 1.30.

Several participants recounted lessons they learnt from specific stories. One participant recounted the story of Shuvai from the module ‘Finding help for your sick child’, which taught the importance of seeking help from the health facility early. This module was developed based on qualitative data showing that several participants expressed belief in Apostolic Faith Healers and would usually consult them before attending the health facility:

‘I learnt that when a child is sick, I should go to the clinic. I learnt that you should take the child to the hospital, not go to the [Apostolic Faith] shrine first or consult other people in the community; they don't know anything about it [SAM]. In the case of Shuvai, she wasted time seeking help elsewhere, the child lost weight and the ribs were sticking out…’ (10027)

Another participant recounted the lessons she learned from a story in the HIV series module:

‘The story about Vimbai made me understand HIV in a better way. Vimbai did not follow the advice she was given at the health facility and her child also got infected with HIV. I learned that if one is tested for HIV and is commenced on ART, and adheres to treatment, you are likely to live a longer and better life…’ (10028)

Several participants highlighted that handouts containing key messages served as a useful reminder. One participant explained that when her child fell sick, her husband blamed her; however, after completing the module on ‘Causes of SAM’ she shared the handout with him, which helped him to understand that she was not to blame:

‘… he realised that kwashiorkor was not only due to lack of food but it can happen even when the food is available and the child is not eating well. Also, that it's not caused by someone inflicting it on the child. From there on our relationship problems were resolved, from reading that information sheet’. (10023)

Games, storytelling, and pictures or graphics were well‐received and an acceptable mode of learning. Getting caregivers to play games during CKT meetings and in module activities reminded them of their own childhood, which stimulated a desire to play with their children. Participants particularly liked games that combined play and education, such as a ‘snakes and ladders’ game which taught lessons on WASH and nutrition, and a ‘jar of stones’ game which illustrated malabsorption of nutrients when a child is sick, leading to malnutrition (Table 2, 1.31–1.32).

#### 3.2.4 Participant behaviour‐change and intervention uptake

All caregivers reported practising what they had learned, even with limited resources. Most participants found the infant and young child feeding (IYCF) modules helpful, including practical lessons on how to use locally available and more easily accessible and affordable foods to meet the recommended nutritional content in each meal (Table 2, 1.33–1.35). One participant highlighted her improved adherence to antiretroviral medication following the HIV modules (Table 2, 1.36), while another described how the HIV modules gave her hope and encouraged her to continue taking care of herself and her child (Table 2, 1.37).

One participant highlighted that she liked the budgeting module because it taught her to save and budget:

‘Budgeting is important otherwise you waste money… Before… I would use all the money at once expecting to get another job. Now I have a small amount for my pocket money which even my husband does not know of. When I get grocery funds, I buy the groceries and keep the change’. (10023)

One participant explained how the modules helped her deal with stigma and become more confident in speaking about her child's illness without shame:

‘The information… they shared with me made me realise that I was not the only one with a malnourished child, and that it is something I should not be embarrassed about’. (10029)

Overall, caregivers expressed that they enjoyed the Tamba‐SAM intervention, and would want to participate again or continue the intervention (Table 2, 1.38–1.40). Several participants reported that they shared the Tamba‐SAM lessons with neighbours, friends or relatives. Some participants reported that they were now encouraging others based on the lessons they had learned on issues such as IYCF, hygiene and health care‐seeking. Other participants expressed a willingness to recommend the intervention to friends:

‘I have always shared with my friends the various lessons I got from being a Tamba SAM participant’. (10004); see also Table 2, 1.41–1.42.

### 3.3 Endline quantitative data

By Week 12, child weight‐for‐age *Z* score, weight‐for‐height *Z* score and mid‐upper arm circumference had significantly increased, and only 12.0% of children had SAM, versus 57.1% at baseline (*p* < 0.001); there were no significant changes in height‐for‐age *Z* score or head circumference (Table 3). There was no significant change in neurodevelopment over 12 weeks, as assessed by the caregiver‐reported ASQ, but both the caregiver and child component of the OMCI significantly increased from baseline, showing improved caregiver–child interaction. Caregivers showed more equitable gender norms attitudes at the end of the programme, although no change in perceived social support. Mental health improved significantly, with a striking reduction in the proportion of women screening positive for high risk of depression, from 11 (39.3%) at baseline to none at endline.

**Table 3:** Change in child and caregiver characteristics between baseline and endline.

Characteristics	Baseline *N* = 28	Endline (Week 12) *N* = 26	Difference (95% CI)	*p* Value
Child				
*Anthropometry*				
WHZ, mean (SD)	−2.1 (1.5)	−1.2 (1.3)	0.9 (0.3−1.5)	0.003
WAZ, mean (SD)	−3.6 (1.7)	−2.9 (1.6)	0.7 (0.3−1.2)	0.001
HAZ, mean (SD)	−3.6 (1.4)	−3.5 (1.0)	0.1 (−0.2 to 0.4)	0.611
MUAC, mm, mean (SD)	118 (15)	129 (13)	11 (6, 15)	<0.001
HCZ, mean (SD)	−1.3 (2.3)	−1.1 (2.3)	0.2 (−0.1 to 0.5)	0.149
Stunted, %	89.3	92.0	2.7 (−2.0 to 10.0)	0.736
Current SAM, %	57.1	12.0	−45.1 (−70.0 to −20.0)	<0.001
Underweight, %	89.3	60.0	−29.3 (−50.0 to −10.0)	0.013
*Ages and stages*				
Communication, mean (SD)	32.9 (20.1)	34.8 (24.1)	2.0 (−3.1 to 7.1)	0.436
Gross motor, mean (SD)	22.5 (18.4)	25.2 (24.6)	2.7 (−5.2 to 10.5)	0.491
Fine motor, mean (SD)	32.9 (17.7)	30.2 (21.7)	−2.7 (−10.7 to 5.4)	0.500
Problem‐solving, mean (SD)	31.1 (15.2)	31.4 (21.0)	0.4 (−6.4 to 7.1)	0.914
Personal social, mean (SD)	38.9 (15.8)	34.0 (22.0)	−5.0 (−11.4 to 1.5)	0.125
*Caregiver‐child interaction*				
OMCI child, mean (SD)	9.0 (5.0)	13.0 (4.6)	4.0 (2.3−5.8)	<0.001
OMCI mother, mean (SD)	26.3 (6.2)	30.9 (5.1)	4.6 (2.1−7.1)	<0.001
*Caregiver*				
Gender norms attitude score; median [IQR]	3.7 [3.5−4.1]	4.0 [3.5−4.2]	0.30 (0.0−0.5)	0.039
Perceived social support score; median [IQR]	3.1 [2.5−3.4]	2.9 [2.6−3.2]	−0.2 (−0.4 to 0.6)	0.949
EPDS score; median [IQR]	11 [7−14]	5 [2−7]	−6.0 (−9.0 to −3.0)	<0.001
High risk for depression, %^a^	39.3	0	−39.0 (−60.0 to −20.0)	<0.001
AUDIT score, median [IQR]	0 [0−0]	0 [0−0]	0 (0−0)	0.346
Household dietary diversity score; median (IQR)^b^	7.5 (6.0−8.0)	8.0 (6.5−9.0)	0.04 (−1.11 to 1.18)	0.949

Abbreviations: AUDIT, Alcohol Use Disorders Identification Test; CI, confidence interval; EPDS, Edinburgh Postnatal Depression Score; HAZ, height‐for‐age *Z* score; HCZ, head circumference for age *Z* score; IQR, interquartile range; MUAC, mid‐upper arm circumference; OMCI, observation of maternal‐child interaction (women: 0‐36, children: 0‐21); SAM, severe acute malnutrition; WAZ, Weight‐for‐age *Z* score; WHZ, Weight‐for‐height *Z* score.

^a^ Defined as an EPDS score of 12 or more, or answering yes to a question about self‐harm;

^b^ Household dietary diversity score (0–16) is based on the number of food groups consumed.

## 4 DISCUSSION

Complicated SAM is a high‐risk condition, and children have an on‐going risk of mortality and relapse after discharge from hospital (Bwakura‐Dangarembizi et al., 2021). Current management strategies do not effectively promote convalescence following hospitalisation and do not address the social determinants underlying SAM. Psychosocial and stimulation interventions improve neurodevelopment, caregiver–child interaction, and maternal depression in various settings (Baker‐Henningham, 2005; Grantham‐McGregor et al., 1980, 1983, 1987; Nahar et al., 2009, 2012, 2015) and are one of the few postdischarge strategies to reduce mortality (Noble et al., 2021). However, there are few available programmes in areas of high SAM prevalence. We codesigned and piloted a low‐cost, complex intervention to enhance child play, caregiver support, income‐generation and household capabilities in peri‐urban Zimbabwe, with the goal of improving caregiver–child interaction to promote holistic recovery following complicated SAM. We show here that the intervention was feasible to deliver through trained lay workers, acceptable to caregivers, and showed benefits for caregiver–child pairs in a mixed‐methods evaluation.

This study demonstrated that it is feasible and acceptable to use a locally developed psychosocial intervention delivered by lay workers. The cohort had several risk factors for mortality, readmission and failed nutritional recovery: over half had non‐oedematous SAM, almost one‐third had HIV, and almost all were stunted (Bwakura‐Dangarembizi et al., 2021). As such, this pilot study targeted an appropriate group of high‐risk peri‐urban children and their caregivers, living in economic precarity with perennial food insecurity and a high prevalence of baseline depression. The intervention was successfully delivered by women who themselves had previously had a child hospitalised with SAM. Lay workers have successfully delivered psychological interventions in multiple contexts, and previous studies highlight the importance of ensuring adequate supervision and frequent retraining to maintain fidelity of intervention delivery (Shahmalak et al., 2019). We achieved this through observed sessions, review of audio‐recorded sessions, and weekly debrief meetings. Our qualitative data show that lay workers were welcomed by participants due to their shared lived experience, and they could deliver all elements of the intervention, suggesting that larger scale rollout using this approach is possible. However, it was interesting that participants preferred to attend clinics for the intervention, rather than receive home visits, due to the lack of space, poor privacy, and potential for stigma and gossip from neighbours and landlords.

It is encouraging that participants reported using the tools and learning imparted during the intervention. Problem‐solving therapy assisted caregivers to find homegrown solutions to dietary problems using items available in the home. Participants enjoyed the group therapy sessions and derived benefits from interaction with other caregivers experiencing similar circumstances. Sharing ideas on income generation resulted in several participants starting small businesses. We found striking improvements in caregiver mental health after 12 weeks. These findings contrast with a study from Bangladesh in which maternal depression was not impacted by a psychosocial intervention (Nahar et al., 2015). It is possible that the overall intervention package, incorporating child stimulation and play together with problem‐solving therapy and peer‐support, is critical in addressing the holistic needs of caregiver–child pairs. Together, these factors likely contribute to the efficacy of psychosocial interventions in improving the outcomes of children with SAM (Noble et al., 2021); however, further study of the mechanism of action at scale will be valuable.

This study had strengths and weaknesses. The intervention was structured around existing evidence‐based interventions, then refined through codesign with caregivers using an iterative approach, to make it contextually relevant. However, in this small pilot study, we could not conclude whether the intervention has efficacy. The study was not powered to detect changes in mortality or neurodevelopment, had no control group, and undertook endline assessments after only 12 weeks; however, there were promising signs of improvement in caregiver–child interaction and changes in caregiver capabilities, which collectively suggest the quality of nurturing care may have improved.

In summary, we report the development and initial evaluation of a codesigned caregiver support and child play intervention aimed at enhancing the recovery of children hospitalised with SAM. This strategy was feasible, acceptable and showed promise in modifying caregiver knowledge, attitudes and practice. This intervention now needs to be tested in a randomised trial to determine whether it improves clinical outcomes, with attention to process indicators to understand the mechanism of action and consideration of sustainability at scale.

## AUTHOR CONTRIBUTIONS

Louisa Mudawarima, Lisa F. Langhaug, Mutsa Bwakura‐Dangarembizi and Andrew J. Prendergast designed the study. Louisa Mudawarima, Mutsa Bwakura‐Dangarembizi, Robert Ntozini and Andrew J. Prendergast secured funding. Louisa Mudawarima, Florence D. Majo, Epiphania Munetsi and Lloyd Dzapasi conducted training and supportive supervision. Jacqueline Kabongo, Florence D. Majo and Anesu Dzikiti conducted the study with oversight from Batsirai Mutasa, Robert Ntozini, Lisa F. Langhaug, Isabella Cordani, Mutsa Bwakura‐Dangarembizi and Andrew J. Prendergast. Jacqueline Kabongo, Joice Tome, Bernard Chasekwa and Robert Ntozini analysed the data. Jacqueline Kabongo, Louisa Mudawarima, Lisa F. Langhaug and Andrew J. Prendergast wrote the paper. All authors reviewed and critically revised the manuscript.

## CONFLICT OF INTEREST STATEMENT

The authors declare no conflict of interest.

## Data Availability

The data that support the findings of this study are available from the corresponding author upon reasonable request.
